# Enhancing Drug Efficacy and Therapeutic Index through Cheminformatics-Based Selection of Small Molecule Binary Weapons That Improve Transporter-Mediated Targeting: A Cytotoxicity System Based on Gemcitabine

**DOI:** 10.3389/fphar.2017.00155

**Published:** 2017-03-27

**Authors:** Justine M. Grixti, Steve O'Hagan, Philip J. Day, Douglas B. Kell

**Affiliations:** ^1^Faculty of Biology, Medicine and Health, University of ManchesterManchester, UK; ^2^Manchester Institute of Biotechnology, University of ManchesterManchester, UK; ^3^School of Chemistry, University of ManchesterManchester, UK; ^4^Centre for Synthetic Biology of Fine and Speciality Chemicals, University of ManchesterManchester, UK

**Keywords:** binary weapon, cheminformatics, gemcitabine, anticancer drugs, pancreatic cancer, drug transporters, phenotypic screening

## Abstract

The transport of drug molecules is mainly determined by the distribution of influx and efflux transporters for which they are substrates. To enable tissue targeting, we sought to develop the idea that we might affect the transporter-mediated disposition of small-molecule drugs via the addition of a second small molecule that of itself had no inhibitory pharmacological effect but that influenced the expression of transporters for the primary drug. We refer to this as a “binary weapon” strategy. The experimental system tested the ability of a molecule that on its own had no cytotoxic effect to increase the toxicity of the nucleoside analog gemcitabine to Panc1 pancreatic cancer cells. An initial phenotypic screen of a 500-member polar drug (fragment) library yielded three “hits.” The structures of 20 of the other 2,000 members of this library suite had a Tanimoto similarity greater than 0.7 to those of the initial hits, and each was itself a hit (the cheminformatics thus providing for a massive enrichment). We chose the top six representatives for further study. They fell into three clusters whose members bore reasonable structural similarities to each other (two were in fact isomers), lending strength to the self-consistency of both our conceptual and experimental strategies. Existing literature had suggested that indole-3-carbinol might play a similar role to that of our fragments, but in our hands it was without effect; nor was it structurally similar to any of our hits. As there was no evidence that the fragments could affect toxicity directly, we looked for effects on transporter transcript levels. In our hands, only the ENT1-3 uptake and ABCC2,3,4,5, and 10 efflux transporters displayed measurable transcripts in Panc1 cultures, along with a ribonucleoside reductase RRM1 known to affect gemcitabine toxicity. Very strikingly, the addition of gemcitabine alone increased the expression of the transcript for ABCC2 (MRP2) by more than 12-fold, and that of RRM1 by more than fourfold, and each of the fragment “hits” served to reverse this. However, an inhibitor of ABCC2 was without significant effect, implying that RRM1 was possibly the more significant player. These effects were somewhat selective for Panc cells. It seems, therefore, that while the effects we measured were here mediated more by efflux than influx transporters, and potentially by other means, the binary weapon idea is hereby fully confirmed: it is indeed possible to find molecules that manipulate the expression of transporters that are involved in the bioactivity of a pharmaceutical drug. This opens up an entirely new area, that of chemical genomics-based drug targeting.

## Introduction

In a typical small molecule drug discovery programme pipeline, candidate (“hit”) compounds for treating a particular disease are selected from a large chemical library, and after various modifications (to form “leads” and variants thereof) enter “phase 1,” a testing for safety at low doses in healthy volunteers. “Attrition” is a term used to describe the failure of such molecules to progress further to market, via phases 2 and 3 (small and larger clinical trials) (Kola and Landis, 2004; Empfield and Leeson, 2010; Leeson and Empfield, 2010; Leeson, 2016). Nowadays attrition occurs largely for reasons of toxicity or lack of efficacy (Kola and Landis, 2004; Arrowsmith and Miller, 2013), and runs in excess of 90% (e.g., Kola and Landis, 2004; Kell, 2013, and see for full details http://csdd.tufts.edu/files/uploads/Tufts_CSDD_briefing_on_RD_cost_study_-_Nov_18,_2014.pdf), with gross pharmacokinetics and pharmacodynamics (as assessed at the whole organ level) being seen as less of an issue than it once was (Kola and Landis, 2004). The simple consequence of this level of attrition is that it costs ~10 times more than it might, per molecule, now as much as $2.5 Bn, to bring a drug successfully to market.

### Role of transporters in cellular drug uptake

We have argued that a lack of understanding of human metabolism and of the transporters necessary to get orally active drugs across intestinal epithelia and into target cells is one of the chief causes of attrition. By now, following a similar programme in yeast (Herrgård et al., 2008), we do have a reasonable model of the human metabolic network (Swainston et al., 2013, 2016; Thiele et al., 2013), with fully one third of the steps involving some kind of transport(er), and with uptake transporters of the SoLute Carrier families (SLCs) (Hediger et al., 2004, 2013) being woefully understudied (César-Razquin et al., 2015). In particular, although it remains underappreciated, we have rehearsed on multiple occasions the abundant evidence that the non-transporter- (i.e., bilayer-) mediated uptake of drugs through intact cell membranes is normally negligible (e.g., Dobson and Kell, 2008; Dobson et al., 2009a,b; Kell et al., 2011, 2013, 2015; Lanthaler et al., 2011; Kell, 2013, 2015a,b, 2016a,b; Kell and Goodacre, 2014; Kell and Oliver, 2014; Mendes et al., 2015; O'Hagan and Kell, 2015a; Kell, 2015a,b), a striking recent example being that of Superti-Furga and colleagues (Winter et al., 2014). This shifts the agenda to one of molecular enzymology and systems biology, in which we need to discover (i) which transporters transport which drugs (Giacomini et al., 2010; Sugiyama and Steffansen, 2013), (ii) their expression profiles in different membranes and tissues, and (iii) their kinetic properties. In other words it leads us to recognize that this is fundamentally a problem of systems pharmacology (e.g., van der Greef and Mcburney, 2005; Berger and Iyengar, 2009; van der Graaf and Benson, 2011; Antman et al., 2012; Rostami-Hodjegan, 2012; Waldman and Terzic, 2012; Zhao and Iyengar, 2012; Kell and Goodacre, 2014; Westerhoff et al., 2015; Kell, 2015a).

### Transporter-mediated drug targeting

A particularly nice example of the overwhelming use of transporters for drug uptake comes from the study of Superti-Furga and colleagues (Winter et al., 2014) using haploid cells and determining that very much less than 1% of sepantronium uptake could have occurred other than via a specific SLC called SLC35F2. In a similar and complementary vein, the expression profile of specific transporters allows one to target drug substrates to the particular tissues in which the relevant transporters are most highly expressed. This has been illustrated beautifully by Pfefferkorn and colleagues for both a glucokinase activator (Pfefferkorn et al., 2012; Pfefferkorn, 2013; Sharma et al., 2015) and a “statin”-type drug (Pfefferkorn et al., 2011) that are both targeted to the liver via proteins of the Organic Anion Transport Protein (OATP/SLCO/SLC21) (Hagenbuch and Stieger, 2013) family. In this case substantial concentration ratios of e.g., hepatocyte: pancreas of 50:1 (Pfefferkorn et al., 2012) and hepatocyte:myocyte of 250,000:1 (Pfefferkorn et al., 2011) could be achieved (a finding hard to explain on the basis of any significant bilayer permeability!). Other examples of tissue-selective drug targeting include a liver-targeted stearoyl desaturase inhibitor (Oballa et al., 2011; Ramtohul et al., 2011; Liu, 2013), various other liver-targeted drugs based on OATPs (Buxhofer-Ausch et al., 2013; Tu et al., 2013), and a prostate-specific targeting of an iodide transporter for radio-iodine-mediated cell killing (Kakinuma et al., 2003).

These examples show what can be achieved in terms of drug targeting if the transporter distribution happens to work to one's advantage “naturally,” but cannot be exploited directly when it does not.

The glucokinase activator case is important, since if such drug molecules were allowed to enter all tissues they proved toxic (Pfefferkorn et al., 2012; Pfefferkorn, 2013). A similar and particular case of interest is that of broadly cytotoxic anticancer drugs, where we evidently need mechanisms to target them solely to the tissue of interest, and where we might then greatly improve their therapeutic index. Since the tissue-dependent expression of such transporter molecules is highly heterogeneous (see e.g., almost any dataset in the human protein atlas http://proteinatlas.org/ Uhlén et al., 2015, including those for SLC28 http://www.proteinatlas.org/search/slc28 and SLC29 http://www.proteinatlas.org/search/slc29), it must be subject to regulation (e.g., Pennycooke et al., 2001; Del Santo et al., 2001; Fernández-Veledo et al., 2004, 2007; Plant, 2016). Thus, just as with the small-molecule-driven induction of pluripotent stem cells (Okita et al., 2007; Feng et al., 2009; Desponts and Ding, 2010; Li and Ding, 2010; Zhang, 2010; Grskovic et al., 2011; Li et al., 2012, 2014; Li X. et al., 2015; Jung et al., 2014; Kang et al., 2014), that regulation can similarly be affected by pharmacological intervention with small molecule effectors. Thus, our aim was to seek small molecules that were themselves without cytotoxic effects but that could increase the response of different target cells to anti-cancer drugs that are otherwise present at only a barely cytotoxic level, in particular by modulating the level of activities of specific uptake transporters. It differs from the use of pairs of existing drugs of known activities (e.g., Borisy et al., 2003; Lehár et al., 2007, 2008, 2009a,b; Zimmermann et al., 2007; Wright, 2016), but, interestingly, bears a clear resemblance to the overall strategy used in traditional Chinese medicine where a “shi” (“courier”) herb is used to assist the delivery of the main ingredient (“Jun” or “Emperor” herb) to its site of action (Zhao et al., 2015). We refer to this combination as a “binary weapon.”

### Gemcitabine and pancreatic cancer

The nucleoside analog gemcitabine (2',2'-difluorodeoxycytidine, Gemzar®) (Alvarellos et al., 2014) is one of the most commonly used chemotherapeutic agents in pancreatic adenocarcinoma, the carcinoma with arguably the least favorable prognosis (5-year survival time) of any (Bhattacharjee et al., 2014; Waddell et al., 2015). Like all nucleoside inhibitors of this type, it must first be transported into the cell and then be metabolized (phosphorylated) to exert its clinical action (thereby lowering its ability to act as a substrate for efflux pumps Fukuda and Schuetz, 2012, though see below). Gemcitabine has multiple intracellular targets, and up-regulation of these targets or nucleoside-metabolizing enzymes such as ribonucleotide reductase (RRM1) may confer resistance to this drug (Bergman et al., 2002, 2005; Nakano et al., 2007; Minami et al., 2015). The main uptake transporters are considered to be ENT1 (SLC29A1) and CNT1/3 (SLC28A1/3) of the SLC28/29 families (Kong et al., 2004; Podgorska et al., 2005; Veltkamp et al., 2008; Young et al., 2008, 2013; Molina-Arcas et al., 2009; Cano-Soldado and Pastor-Anglada, 2012; Molina-Arcas and Pastor-Anglada, 2013) (Table 1). SLC28 transporters are sodium-dependent concentrative nucleoside transporters (Smith et al., 2007), while SLC29 are equilibrative. Notably, there is considerable evidence that the potency (cytotoxicity) of gemcitabine is strongly related to the expression level(s) of these transporters (e.g., Burke et al., 1998; Mackey et al., 1998a,b; Baldwin et al., 1999; Rauchwerger et al., 2000; Cass, 2001; Achiwa et al., 2004; Spratlin et al., 2004; Giovannetti et al., 2005, 2006, 2007; King et al., 2006; Marcé et al., 2006; Mey et al., 2006; Mini et al., 2006; Leung and Tse, 2007; Mori et al., 2007; Oguri et al., 2007; Zhang et al., 2007; Cano-Soldado et al., 2008; Molina-Arcas et al., 2008; Pérez-Torras et al., 2008; Veltkamp et al., 2008; Andersson et al., 2009; Damaraju et al., 2009; Farrell et al., 2009, 2016; Köse and Schiedel, 2009; Maréchal et al., 2009, 2012; Wong et al., 2009; Hagmann et al., 2010; Lane et al., 2010; Molina-Arcas and Pastor-Anglada, 2010; Okazaki et al., 2010; Paproski et al., 2010, 2013; Santini et al., 2010, 2011; Spratlin and Mackey, 2010; Tanaka et al., 2010; Bhutia et al., 2011; De Pas et al., 2011; Gusella et al., 2011; Komori et al., 2011; Matsumura et al., 2011; Borbath et al., 2012; Choi, 2012; Gesto et al., 2012; Kobayashi et al., 2012; Koczor et al., 2012; Morinaga et al., 2012; Murata et al., 2012; Eto et al., 2013; Nakagawa et al., 2013; Skrypek et al., 2013; Xiao et al., 2013; Chan et al., 2014; Deng et al., 2014; Greenhalf et al., 2014; Khatri et al., 2014; Koay et al., 2014; Lee et al., 2014; Lemstrová et al., 2014; Liu et al., 2014; Nordh et al., 2014; Tavano et al., 2014; Wu et al., 2014; de Sousa Cavalcante and Monteiro, 2014; Hung et al., 2015; Pastor-Anglada and Pérez-Torras, 2015; Yamada et al., 2016).

**Table 1:** **The main human nucleoside transporters and some of their properties**.

**Name**	**Notes**	**Nucleosides transported**			
		**Adenosine**	**Thymidine**	**Cytidine**	**Guanosine**
ENT1/SLC29A1	Transports adenosine, guanosine, inosine, uridine, cytidine, thymidine with K_m_-values ranging from 50 to 580 μM (You and Morris, 2014)	High affinity (Aran and Plagemann, 1992; Mackey et al., 1998b; Ward et al., 2000; Vickers et al., 2002; Spratlin et al., 2004)	High affinity (Aran and Plagemann, 1992; Mackey et al., 1998b; Ward et al., 2000; Vickers et al., 2002; Spratlin et al., 2004)	High affinity (Aran and Plagemann, 1992; Mackey et al., 1998b; Ward et al., 2000; Vickers et al., 2002; Spratlin et al., 2004)	High affinity (Aran and Plagemann, 1992; Mackey et al., 1998b; Ward et al., 2000; Spratlin et al., 2004)
ENT2/SLC29A2	To date, hENT2 (and intracellular hENT3) are the first discovered, and so far only identified, transporter proteins for nucleobases inside human cells and tissues (Yao et al., 2011)	hENT2 is a generally low affinity nucleoside transporter with 2.6-, 2.8-, 7.7-, and 19.3-fold lower affinity than hENT1 for thymidine, adenosine, cytidine, and guanosine, respectively (Aran and Plagemann, 1992; Mackey et al., 1998b; Ward et al., 2000; Spratlin et al., 2004; You and Morris, 2014). High affinity toward adenosine metabolites (inosine and hypoxanthine) (Ward et al., 2000). 4-fold higher toward inosine (Aran and Plagemann, 1992; Ward et al., 2000; Vickers et al., 2002; Spratlin et al., 2004; You and Morris, 2014)			
ENT3/SLC29A3	Also carries purines and pyrimidines nucleosides but functions predominantly in intracellular membranes (Baldwin et al., 2005; Yao et al., 2011)	Low affinity (Baldwin et al., 2005). Transport exhibits a pH optimum (Baldwin et al., 2005; You and Morris, 2014)		Also transports adenine (Yao et al., 2011; You and Morris, 2014)	
CNT1/SLC28A1	Pyrimidine selective (Ward et al., 2000; Ritzel et al., 2001; You and Morris, 2014)			Also transports adenosine with high affinity (Ritzel et al., 2001)	
CNT2/SLC28A2	Purine selective (Loewen et al., 1999; Wang and Giacomini, 1999; Ward et al., 2000; Saunders et al., 2011)	High affinity for purines (K_m_ < 10 μM) (Cansev, 2006; Huber-Ruano et al., 2010; You and Morris, 2014)	Low affinity for pyrimidines? (Cansev, 2006)		
CNT3/SLC28A3	Non-selective for purine and pyrimidine (Ward et al., 2000; You and Morris, 2014)				

*SLC29 family and concentrative and Na^+^-coupled, while SLC28 family is equilibrative. ENT4 also exists but seems to play little or any role*.

ABC-type efflux transporters are heavily involved in drug resistance in both mammals (e.g., Liu et al., 2005; Fukuda and Schuetz, 2012; Rosenberg et al., 2015; Silva et al., 2015) and microbes (e.g., Putman et al., 2000; Du et al., 2014; Prasad and Rawal, 2014; Li X.-Z. et al., 2015), and may be of value in industrial biotechnology (Kell et al., 2015). Gemcitabine may also be a substrate for certain efflux transporters such as ABCG2/BRCP (König et al., 2005; Keppler, 2011; Chen et al., 2012; Lemstrová et al., 2014), although “knockdown of ABCC3, ABCC5 or ABCC10 individually did not significantly increase gemcitabine sensitivity” (Rudin et al., 2011). Finally, gemcitabine may also be deaminated in plasma, leading to its clearance (Hodge et al., 2011).

Other small molecules known to affect the response of pancreatic cancer cells to gemcitabine include nicotine (Banerjee et al., 2013, 2014), while molecules that affect nucleoside transporter expression include bile acids (Klein et al., 2009). Finally, erlotinib, gefitinib, and vandetanib inhibit human nucleoside transporters and thereby protect cancer cells from gemcitabine cytotoxicity (Damaraju et al., 2014), while a variety of kinase inhibitors (Huang et al., 2002, 2003, 2004) and dihydropyridine-type calcium channel antagonists (Li et al., 2007) may also affect nucleoside transport.

In particular, however, and not least since the small molecule indole-3-carbinol (which is probably converted to 3,3′-diindolylmethane Banerjee et al., 2009) had been stated to increase both ENT1 expression and the sensitivity of pancreatic carcinoma cells to gemcitabine (Wang et al., 2011), possibly acting via miRNA-21 (Giovannetti et al., 2010; Hwang et al., 2010; Melkamu et al., 2010; Paik et al., 2013), as too did the molecule “S-1” (Nakahira et al., 2008; Jordheim and Dumontet, 2013), the gemcitabine/nucleoside transporter system seemed ideal for the test of our “binary weapon” strategy. The present paper reports the results of this approach.

## Materials and methods

### Cells and reagents

The human pancreatic duct epithelioid carcinoma cell line, Panc1 (see Gou et al., 2007), and the human embryonic kidney cell line, HEK293 were grown in Dulbecco's modified Eagle's medium (DMEM) (Sigma). The human bone marrow neuroblastoma cell line, SH-SY5Y was grown in a 1:1 mixture of Eagle's Minimum Essential Medium (Sigma) and F12 Medium (Sigma). All cell culture media were supplemented with 10% heat-inactivated fetal bovine serum (FBS), 200 mM L-glutamine, and a 5 mL solution containing 10,000 units.mL-1 penicillin and 10 mg.mL-1 streptomycin. The immortal human pancreatic duct epithelial cell line, hPDE was grown in Keratinocyte-SFM (1X) medium (ThermoFisher), supplemented with 10 mg.mL-1 streptomycin. All four cell lines were obtained and karyotyped locally. Cells were routinely maintained at 37°C in a humidified 5% CO2 atmosphere, in continuous exponential growth at a cell density ranging between 1 × 10^5^ and 1 × 10^6^ cells.mL-1, by passaging every 3 or 4 days. Cell line authenticity was confirmed through karyotype testing (University of Manchester, UK).

### Cell growth/viability assay

Cells were seeded in a 96-well plate at a density of 5,000 cells/well, in triplicate, and left to attach. Gemcitabine, present at different concentrations, was added directly to the cells, and left to incubate for an additional 96 h. Cells were then subjected to the MTT Cell Proliferation Assay as per the manufacturer's instructions (Sigma). Absorbance at 570 nm was measured 3 h after the addition of 10 μL of MTT salt reagent/well.

### Maybridge fragment screening

Maybridge fragments (MBFs) obeying the “rule of three” (Congreve et al., 2003) were supplied at 100 mM in DMSO and were deployed into the assay plates using an ECHO contactless liquid handler (Labcyte, Inc). For screening purposes, the first 500 MBFs (Library 1) were pooled, i.e., each well in a 96-well plate had a pool of six MBFs. Cells were seeded in a 96-well plate at a density of 5,000 cells/well, in triplicate, and left to attach overnight. Following incubation, the growth medium was replaced with fresh medium containing the pooled MBFs, each fragment present at 10 μM, followed by an additional 24 h incubation. The cells were further incubated with the fragments in the presence of gemcitabine at 20 nM for 96 h. Cells were then subjected to the MTT Cell Proliferation Assay as described above.

To study the effect of each MBF on its own rather than in a pool, the candidate pooled fragments (i.e., showing activity) were de-convolved, i.e., one MBF/well, and cells were plated and treated as described above.

### Specificity experiments

SH-SY5Y cells were seeded at a density of 12,500 cells/well, HEK293 and hPDE cells at a density of 10,000 cells/well, in triplicate in a 96-well plate, and left to attach overnight. Following incubation, the medium was replaced with fresh medium containing the MBF hits (i.e., MBF D1, B1, 10, 11, 12, and 20) at 10 μM, followed by an additional 24 h incubation period. Cells were further incubated with the fragments in the presence of gemcitabine at 100 nM for 72 h (SH-SY5Y cells) and 96 h (HEK293 and hPDE cells). Cell viability was then assessed using the MTT Cell Proliferation Assay.

### Cheminformatic analyses

These were all performed as in our previous work of this type (O'Hagan and Kell, 2015a,b,c; O'Hagan et al., 2015; O'Hagan and Kell, 2016), using the KNIME workflow system (see e.g., Berthold et al., 2008; Mazanetz et al., 2012; Meinl et al., 2012; Warr, 2012; O'Hagan and Kell, 2015b and http://knime.org/).

### Maybridge fragment titration experiments

Cells were seeded in a 96-well plate at a density of 5,000 cells/well, in triplicate, and left to attach overnight. Following incubation, the medium was replaced with fresh medium containing MBFs at different concentrations (3, 10, 30, 100, and 300 μM) followed by an additional 24 h incubation. Cells were further incubated with the fragments in the presence of gemcitabine at 100 nM for 96 h. Cells were then assessed using the MTT Cell Proliferation Assay as described earlier.

### Cell culture treatments for gene dysregulation studies

To examine the effect of gemcitabine and MBFs, alone or in combination; on expression of the influx and efflux transporter genes and of the RRM1 gene, cells were seeded in a 6-well plate at a density of 30,000 cells/well, in duplicate, and left to attach overnight. Following incubation, in studies where the effects of the MBFs alone were studied, the medium was replaced with fresh medium containing MBFs at 10 μM, followed by further incubation for 24 h. For studies where the effects of gemcitabine alone were studied, the medium was replaced with fresh medium containing gemcitabine at 100 nM, followed by further incubation for 96 h. For studies in which cells were treated with gemcitabine in combination with the fragments, the cells were first pre-treated with MBFs at 10 μM for 24 h, followed by further incubation with the fragments at 10 μM in the presence of gemcitabine at 100 nM for 96 h. Cells were harvested using TRIzol® reagent (Life Technologies) and stored in −80°C until use.

### Total RNA isolation and quantitative real-time reverse transcription polymerase chain reaction (RT-qPCR)

Following treatment as described above, total cellular RNA was isolated from the cells using the RNeasy isolation kit (Qiagen) according to the manufacturer's instructions. RNA concentration was determined using a NanoDrop® Spectrophotometer (NanoDrop ND-1000, NanoDrop Technologies, Wilmington, USA). The OD_260/280 nm ratios of all RNA samples were determined to be between 1.9 and 2.0, suggesting that all RNA samples were highly pure. RNA integrity was verified by the Agilent RNA 6000 Nano assay kit (Agilent Bioanalyser 2100, Agilent Technologies, Cheadle, UK) as described by the manufacturer. Single-strand cDNA used for RT-qPCR analyses was synthesized from purified total RNA using SuperScript® III Reverse Transcriptase (Life Technologies, Paisley, UK). RT-qPCR were performed using 384-well plates, with a final volume of 10 μL in each well, consisting of 4 μL of cDNA, 5 μL of 2x SYBR Green LightCycler 480_TM PCR master mix (Roche Life Sciences), 0.8 μL of sterile distilled water, 0.1 μL each of 20 μM reverse and forward primers. Samples were performed in triplicates. In the no template controls (negative controls) 4 μL of H_2_O were added, instead of the cDNA samples. RT-qPCR reactions were carried-out using the Roche LightCycler LC_480-qPCR platform, where fluorescence signals were measured in real-time. The protocol, set-up with thermal cycling conditions, consisted of one cycle at 95°C for 10 min, followed by 45 cycles of amplification at 95°C for 10 s, and 60°C for 30 s. Roche LightCycler Data Analysis Software was used to determine the melt curve data as well as the quantification cycle values (Cq values). The changes in expression levels were normalized against two reference gene as determined via GeNorm (REF), and the relative mRNA levels of genes following treatment were calculated using “The Comparative C_T_ Method” (ΔΔC_T_ Method).

### Design of primers for RT-qPCR

The National Centre for Biotechnology Information (NCBI) website (http://www.ncbi.nlm.nih.gov/) was used to identify and obtain mRNA sequences. Exon boundaries were determined from the “European Molecular Biology Laboratories” website (http://www.ensembl.org). This procedure was performed until sets of primers were selected for each target gene. The final step involved checking the primers for similarity using NCBI BLAST (Basic Local Alignment Search Tool) (http://www.ncbi.nlm.nih.gov/BLAST), reducing the chance of primers binding non-specifically.

### Identification of reference genes for RT-qPCR analysis

Samples were analyzed for the expression of each of eight candidate reference genes, namely: ACTB (Beta-Actin), B2M (Beta-2-microglobulin), GAPDH (glyceraldehyde-3-phosphate dehydrogenase), HMBS (hydroxymethyl-bilane synthase), HPRT1 (hypoxanthine phosphoribosyl transferase 1), RPL13A (ribosomal protein L13a), RPL32 (ribosomal protein L32), SDHA (succinate dehydrogenase complex, subunit A) as recommended by Vandesompele et al. (2002). RT-qPCR was performed as described previously, using the primers specific for each candidate reference gene. The GeNorm algorithm software package was used to determine the two most stable reference genes from the set of tested candidate genes by calculating a gene normalization factor, eliminating the least stable genes until a stability value (M) of 0.4 or less was reached (Vandesompele et al., 2002).

## Results

### Effects of gemcitabine and drug fragments on the viability of Panc-1 cells

A standard strategy is to choose a series of molecules that cover chemical space effectively, and for this we chose initially the main Maybridge drug fragment library. It consists of 500 rule-of-three-compliant (Congreve et al., 2003) polar molecules that cover chemical space widely, and where the molecular properties include molecular weight <300, number of hydrogen bond donors ≤3, number of hydrogen bond acceptors ≤3, ClogP ≤3, and in addition, the number of rotatable bonds ≤3 and the polar surface area ≤60Å^2^. While the use of fragments is commonplace in target-based assays, especially where structures are known (e.g., Erlanson and Hansen, 2004; Erlanson et al., 2004; Rees et al., 2004; Carr et al., 2005; Alex and Flocco, 2007; Ciulli and Abell, 2007; Jhoti, 2007; Jhoti et al., 2007; Hubbard, 2008; Fischer and Hubbard, 2009; Schulz and Hubbard, 2009; Whittaker et al., 2010; Leach and Hann, 2011; Erlanson, 2012; Caliandro et al., 2013), we here prefer the use of the rather more successful phenotypic screens (Swinney and Anthony, 2011; Swinney, 2013). Although it is hard to find published examples of phenotypic screens that used fragment-based libraries, we merely point out that 25% of successful (marketed) drugs are no larger than fragments (i.e., <300 Da) (O'Hagan and Kell, 2015c). The fragment-based approach also has the advantage of avoiding the increasing “molecular obesity” (Hann, 2011; Meanwell, 2011) that is seen in some cases as inimical to the finding of successful drugs (Leeson and Springthorpe, 2007; Leeson and Empfield, 2010).

**Figure 1:**
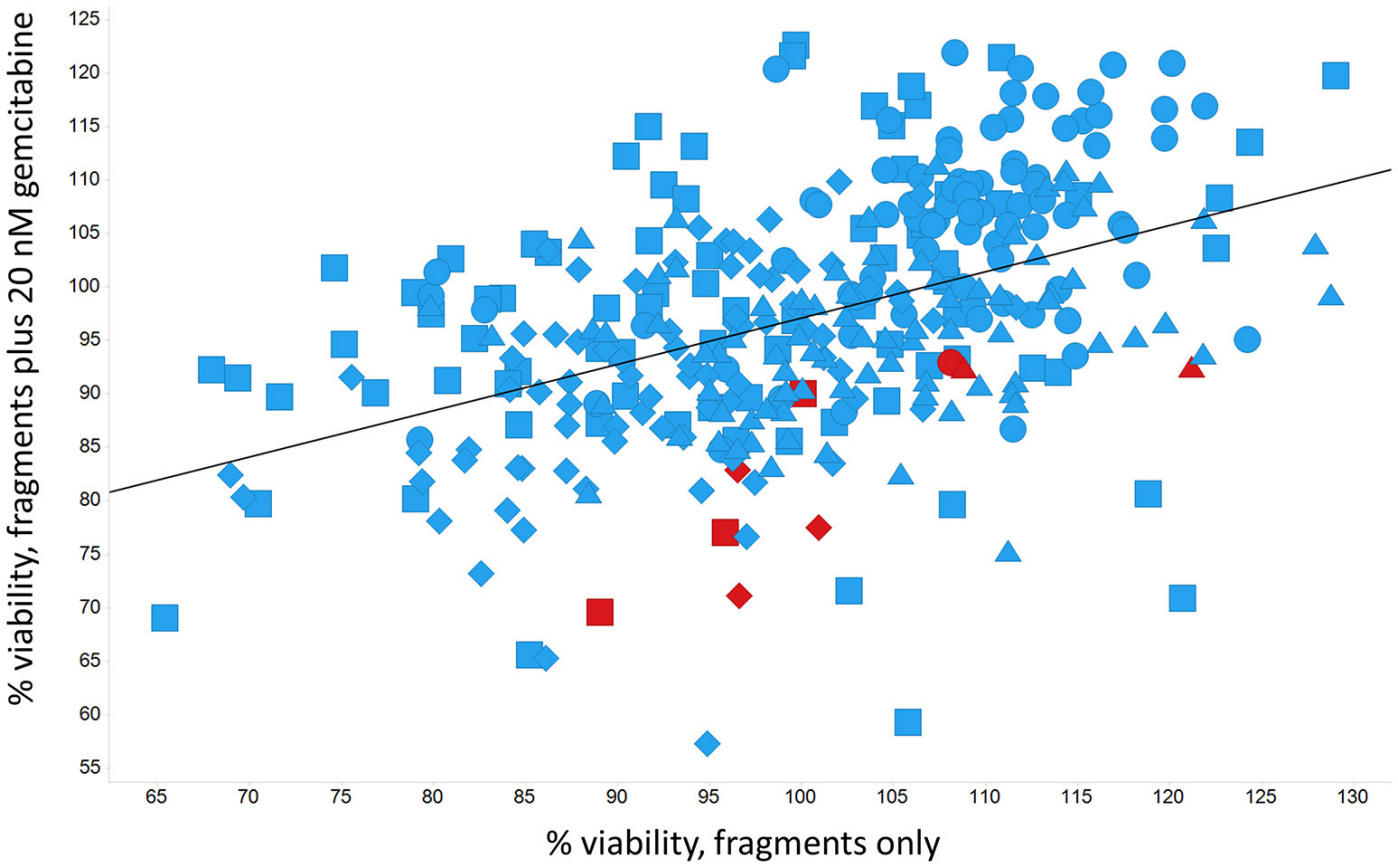
**Effect of 500 Maybridge fragments on the viability of Panc1 cells in the absence and presence of 20 nM gemcitabine**. Experiment number is encoded by shape. Fragments were added in pools of 6. Pools in which there was a hit relative to the same control are marked in red. The line is a line of best fit.

**Figure 2:**
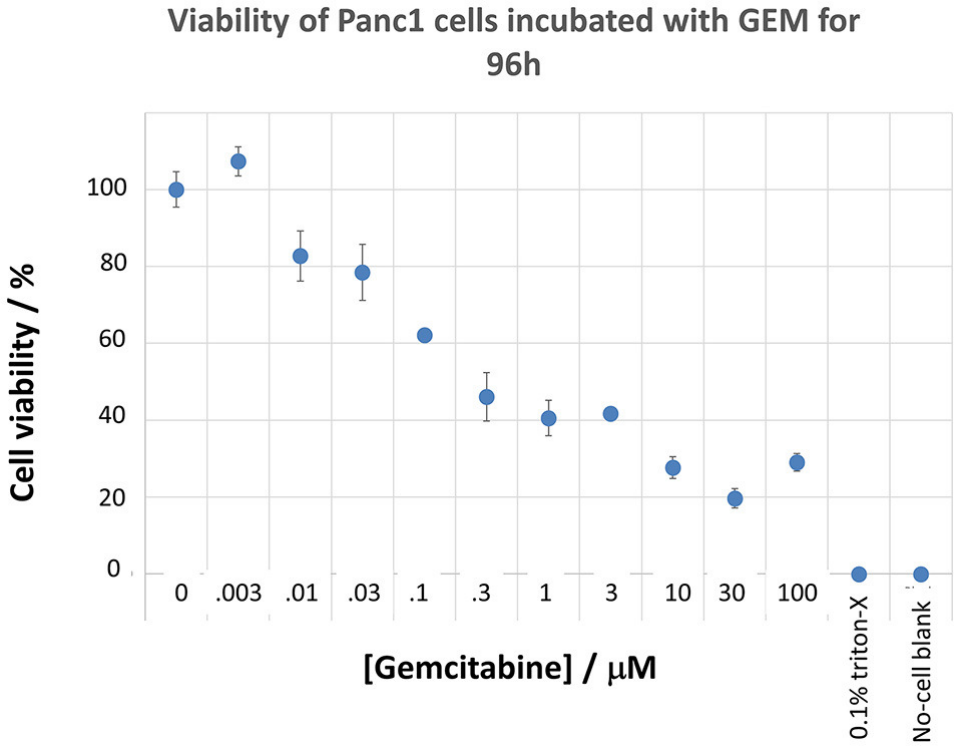
**Effect of gemcitabine concentration on the viability of Panc1 cells**. Cells were grown and pre-incubated with the stated concentration of gemcitabine, and their viability was assessed, as described in the Methods section.

**Figure 3:**
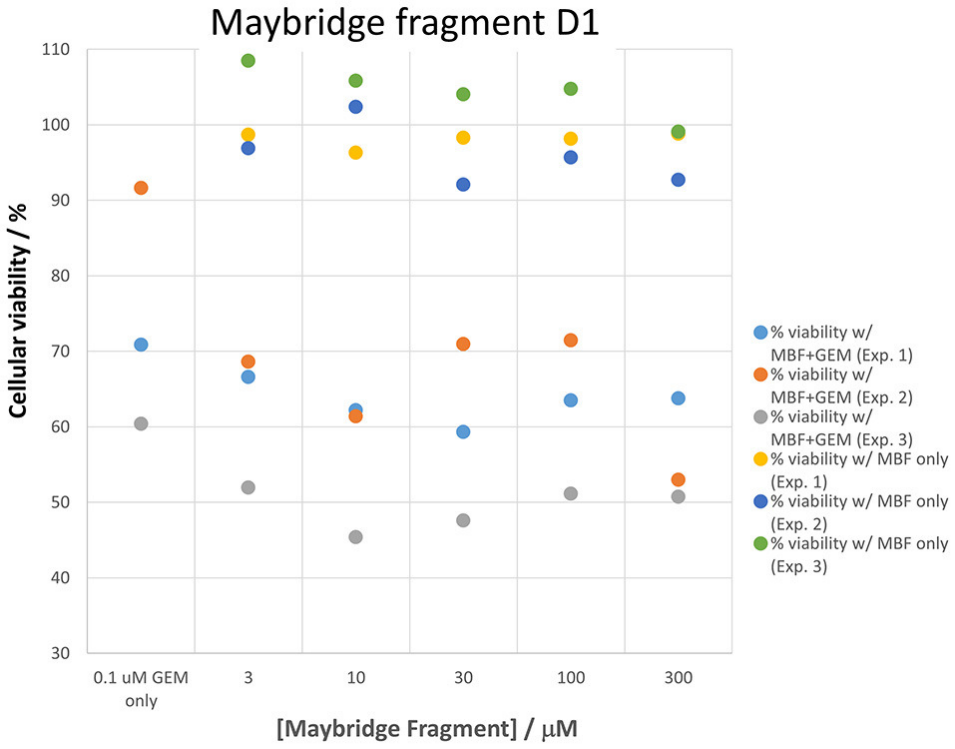
**Variability in gemcitabine sensitivity and the effect of a “hit” (fragment D1) on cellular viability when measured on three sets of cells in cultures grown on different days**. The differences between gemcitabine and gemcitabine plus all “hit” fragments such as D1 is statistically significant at the *P* < 0.05 level (*n* = 3).

**Figure 4:**
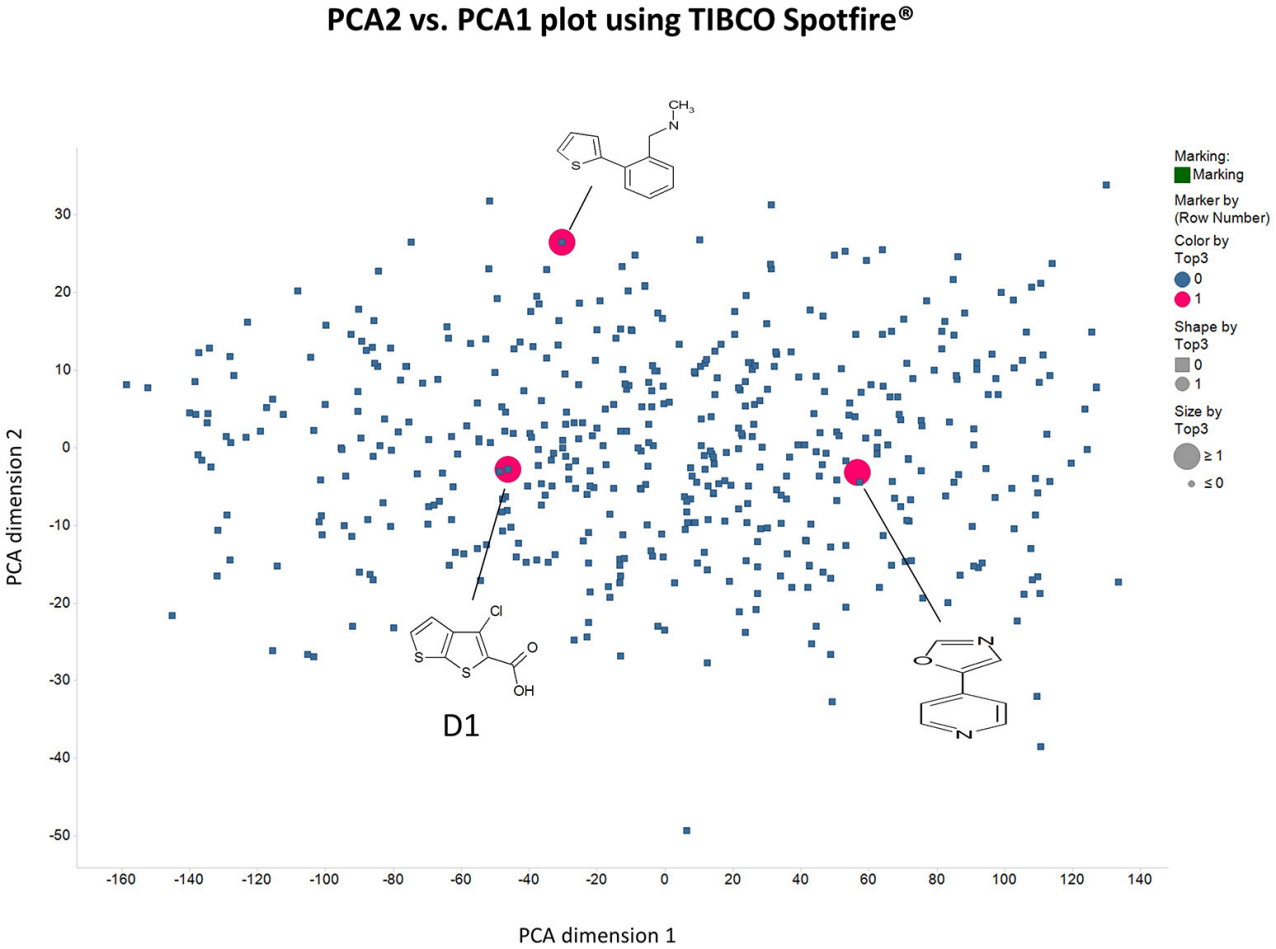
**Distribution in chemical space of the first 500 Maybridge fragments as judged using the principal components of the variance in a set of their biophysical properties (see Methods) as produced using RDKit in KNIME**.

**Figure 5:**
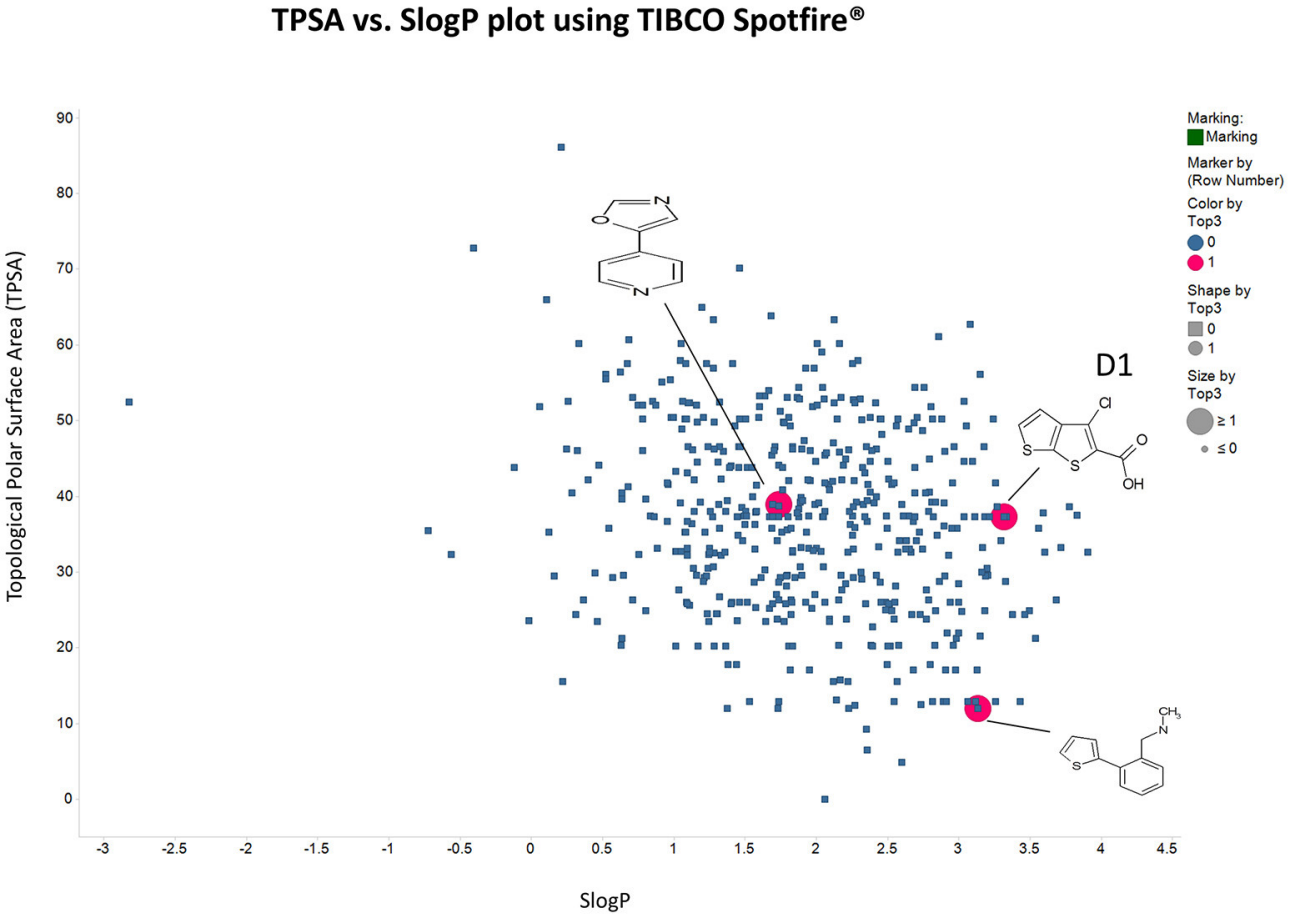
**As in Figure **4** save that the axes are Total Polar Surface area and S log P as calculated using RDKit**.

Panc1 cells are a pancreatic cancer cell line (e.g., Gradiz et al., 2016). Figure 1 shows four separate experiments in which the effect of the pools of the Maybridge fragments (6 at a time) on the viability of cells was assessed in the presence and absence of 20 nM gemcitabine, pointing up three pools containing “hits” (which occurred in at least 3 experiments; there are a total of 336 experiments here). Figure 2 shows the % viability of one set of Panc1 cells as a function of the gemcitabine concentration, as a result of which we later chose 100 nM gemcitabine to assess the efficacy of the individual fragments in increasing its toxicity. Figure 3 shows a titration curve for three repeats with one of the “hits,” the plot also serving to illustrate the variability of the toxicity of gemcitabine alone on different days. Figures 4, 5 show the distribution in chemical space of all 500 fragments in the first Maybridge library and three “hits” at 10 μM that lowered the viability of cells by at least 10% in the presence, but not the absence, of 100 nM gemcitabine. These were retested singly, then together pairwise, resulting in three hits, viz B1, D1, and B12. B12 seemed to interfere with the other two fragments by binding to them directly (UV evidence) and was not used further. Note that a significant issue is that although for a given batch of Panc1 cells the titration curves were reasonably reproducible, they were considerably less so between batches (for reasons that will become apparent below). This meant that each culture had to be used as its own control, as we did e.g., in Figure 2. Another interesting feature was that quite a significant fraction of the fragments (as in Figure 1, and see below) were even somewhat stimulatory to cell growth in the absence of gemcitabine.

**Figure 6:**
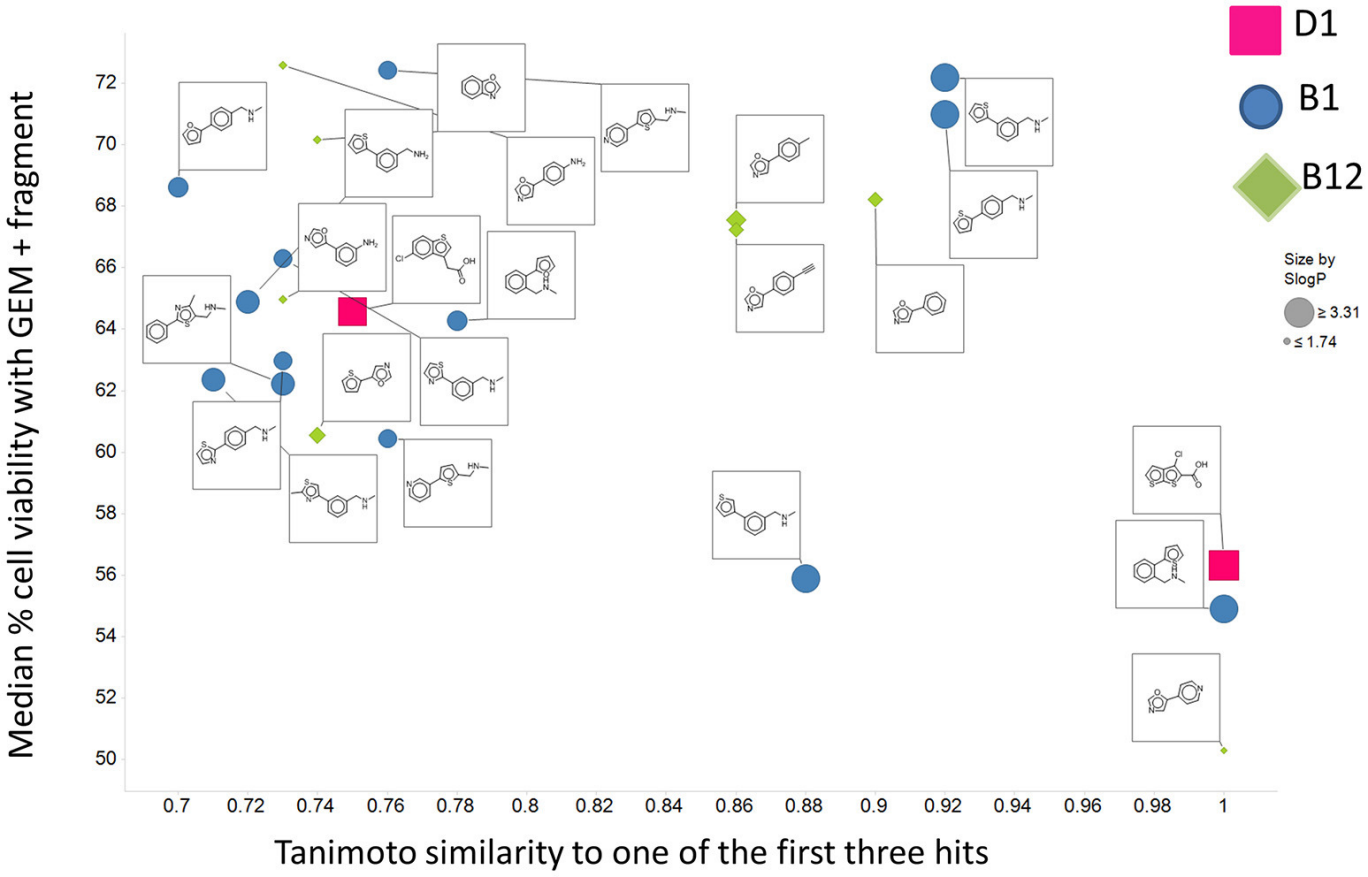
**Tanimoto similarity to the set of three hits in the first 500 Maybridge fragments of 20 molecules selected from the other four libraries**. The average % viability of the cells in the presence of gemcitabine but the absence of Maybridge fragments in this experiment was 81. The starting fragment to which the molecule was most similar is encoded by shape and color, while the S log *P*-value is encoded by size.

There are four other Maybridge fragment libraries of 500 molecules each, covering broadly the same chemical space but in more detail (O'Hagan and Kell, 2015c), and we performed a cheminformatics analysis (MACCS encoding, Tanimoto similarity) to establish which other molecules might be similar, exactly as per the analyses in (O'Hagan et al., 2015). Some 20 molecules had a Tanimoto similarity within 0.7 of one of the three remaining hits and were tested. In this case, the starting % viability was much higher than those in Figure 2. All 20 of these fragments are in fact active, which shows that these molecules (Figure 6) exhibit a very considerable enrichment over the whole library, and illustrates the utility of the principle of molecular similarity (Gasteiger, 2003; Bender and Glen, 2004; Stumpfe and Bajorath, 2011; Maggiora et al., 2014). The figure also illustrates which of the original three hits the new hits are closest to, and encodes their S log *P*-values as the size of the marker. This enormous cheminformatics-based enrichment also gives considerable confidence in our strategy, despite the variability in sensitivity of the Panc1 cells to gemcitabine alone, since such a huge enrichment could not conceivable occur for molecules that were not active. Although none was quite as active as the original hits, all exhibited some kind of activity (Figure 2) (the starting viabilities for two different experiments in the presence of gemcitabine only were 78 and 84%). Of all of these, the seven most potent molecules exhibited activity at 3 μM. One was rather expensive and was again excluded. Thus, we had a total of 6 hits to consider [two from library 1 (B1 and D1), and a total of four from the other four libraries, referred to as fragments 10, 11, 12, and 20]. Table 2 gives their names, SMILES encodings and 2D structures, along with that of indole-3-carboxylic acid (see later).

**Table 2:** **Six hits in the “binary weapon” assay given in three formats, plus indole-3-carbinol**.

**MBF**	**SMILES**	**Name**	
D1	OC(= O)c1sc2sccc2c1Cl	3-chlorothieno[2,3-b]thiophene-2-carboxylic acid	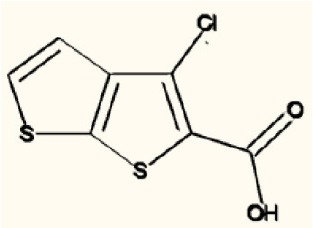
B1	CNCc1ccccc1c2cccs2	N-methyl-N-(2-thien-2-ylbenzyl)amine	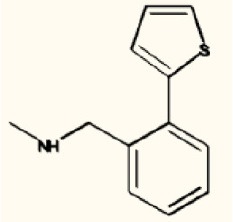
10	S1C(= CC = C1CNC)c1cccnc1	N-methyl-(5-pyrid-3-ylthien-2-yl)methylamine	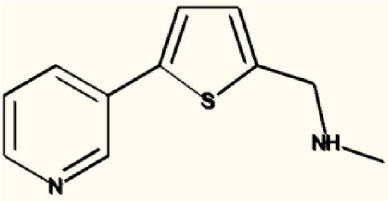
11	S1C(= CC = C1CNC)c1ccncc1	N-methyl-(5-pyrid-4-ylthien-2-yl)methylamine	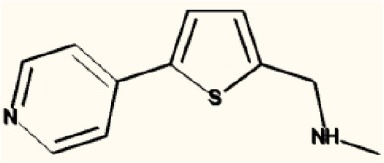
12	S1C = C(c2c1ccc(c2)Cl)CC(= O)O	2-(5-Chlorobenzo[b]thiophen-3-yl)acetic acid	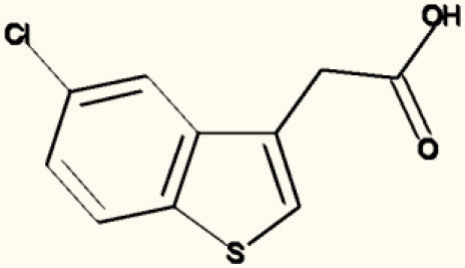
20	N1 = COC(= C1)c1ccc(cc1)N	4-(1, 3-Oxazol-5-yl)aniline	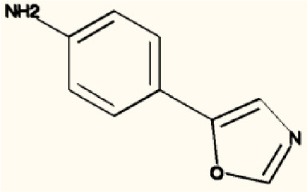
I3C	C1 = CC = C2C(= C1)C(= CN2)CO	Indole-3-carbinol	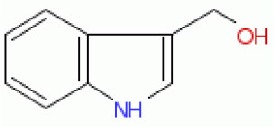

**Figure 7:**
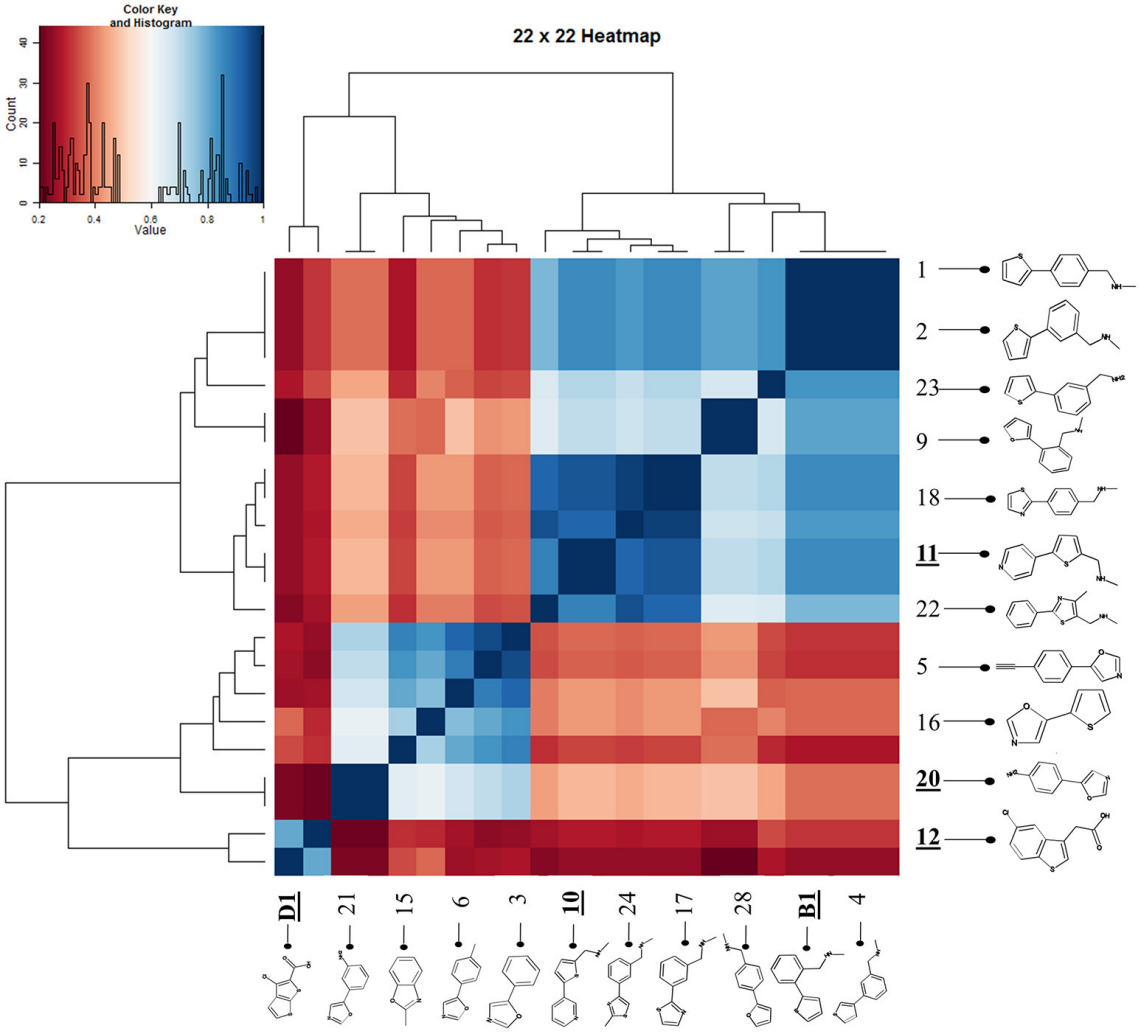
**Chemical similarities of the various hits to each other, and effect of Maybridge fragment 10 on cell viability**. A heatmap showing the three clusters of molecules that could be observed.

**Figure 8:**
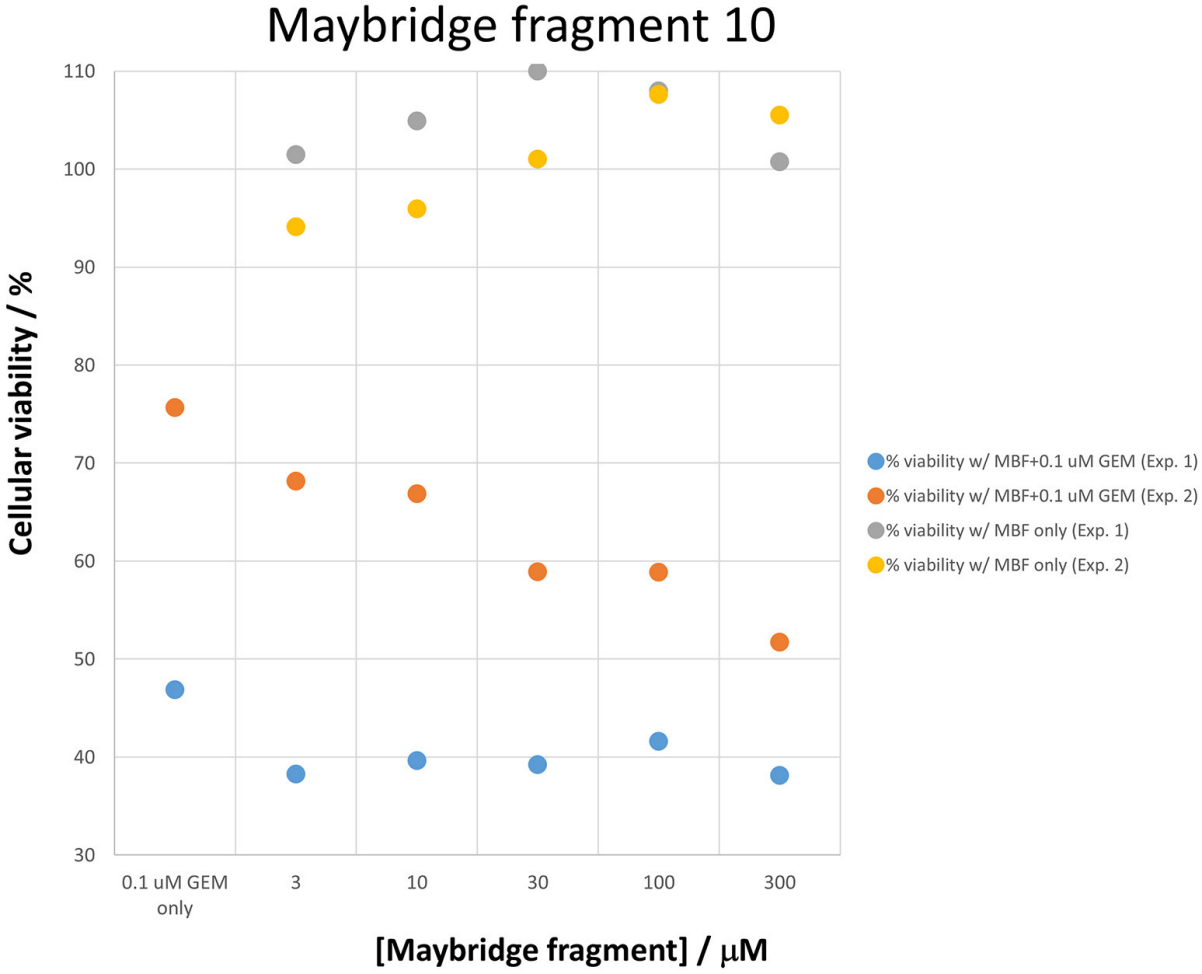
**Two experiments illustrating the effect of Maybridge fragment 10 on cell viability in the absence and presence of gemcitabine, again showing the stimulation in the absence of gemcitabine**. The differences between gemcitabine and gemcitabine plus fragment 10 is statistically significant at the *P* < 0.05 level (*n* = 3).

**Figure 9:**
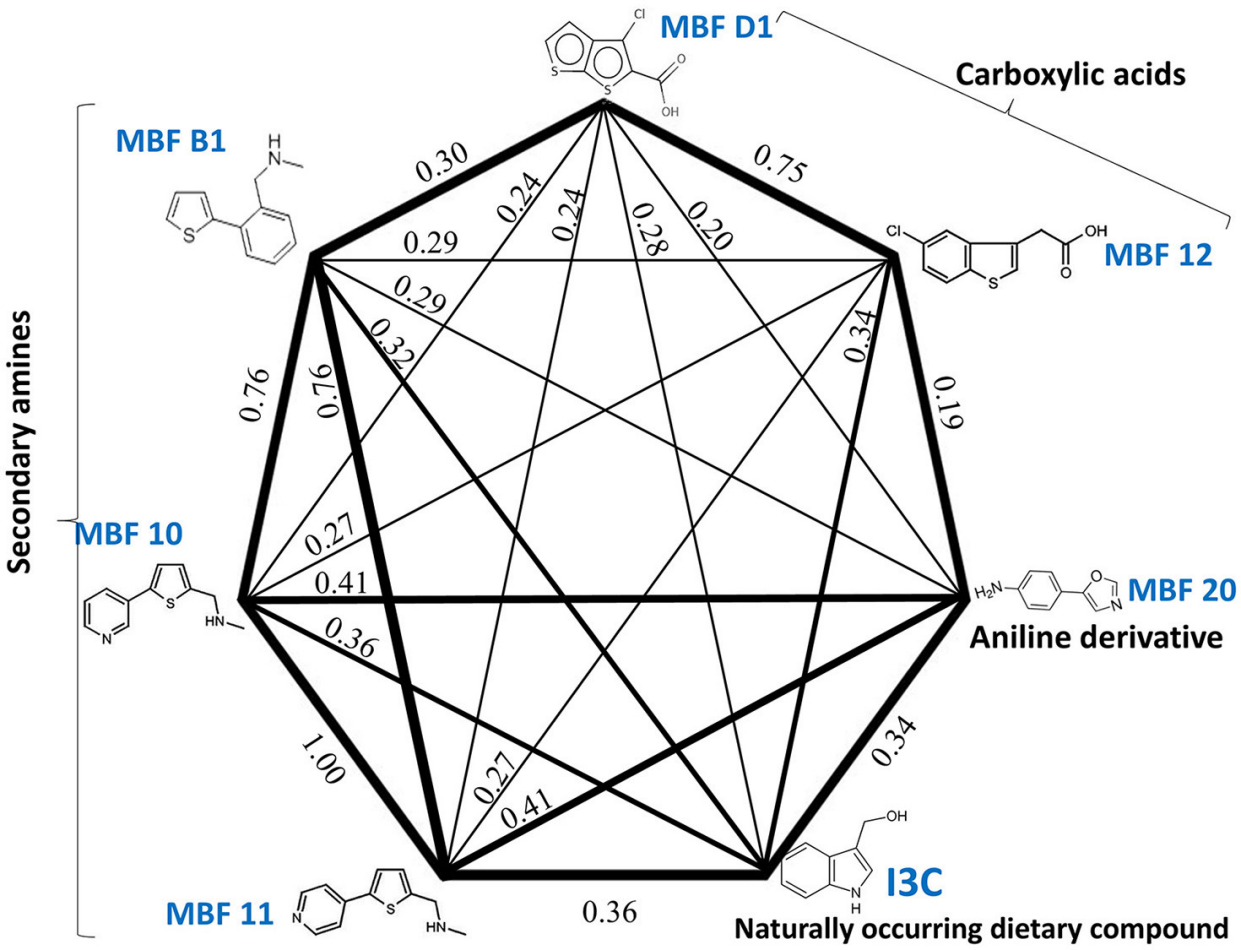
**Tanimoto similarities of the main hits in the three clusters of Figure **7** (plus I3C)**.

Figure 7 shows a (symmetrical) heatmap (MACCS encoding) of the Tanimoto similarities of the 22 most potent molecules, where it can again be seen that the hits are in three clusters. These are B1, 10, and 11 (all are amines), D1 and 12 (carboxylic acids), and 20 (an aniline derivative—possibly to be avoided Benigni and Passerini, 2002; Benigni et al., 2009; Franke et al., 2010). One implication is that they each have different targets (probably plural) but attempts even to show additivity, let alone synergy, met with failure, possibly because the molecules were indeed rather similar to each other in terms of the larger chemical space. Figure 8—equivalent to Figure 3—shows data for two experiments with fragment 10, again illustrating the stimulation of growth by the fragment alone, and its inhibition in the presence of a relatively weakly inhibiting concentration of gemcitabine. Finally, Figure 9 shows the Tanimoto similarities (TS, based on the MACCS encoding) between the six hits plus Indole-3-carbinol (I3C, see below). Fragments within a group showed a Tanimoto similarity of 0.75 or greater, while those between groups were less than 0.5. I3C was not really similar to any of the hits; its highest TS to any of the hits was 0.36. It is especially gratifying to note that MBF10 and MBF11 were both selected and had a TS to each other of 1, as they are in fact structural isomers. Along with the other clusterings, this adds considerable weight to the validity of our assays.

### Effect of indole-3-carbinol on gemcitabine toxicity

Cruciferous vegetables such as *Brassica* spp. are considered to have certain anticancer properties (Higdon et al., 2007; Juge et al., 2007; Fujioka et al., 2016b), and small molecules derived from the hydrolysis of glucosinolates, such as sulforaphane and indole-3-carbinol (I3C), have been implicated in a variety of anticarcinogenic mechanisms (e.g., Chen et al., 2014; Fujioka et al., 2016a). I3C is a small molecule (MW 147.17, well within the range of “fragments”), and Lyn-Cook and colleagues (Lyn-Cook et al., 2010; Wang et al., 2011; Paik et al., 2013) have published that I3C can enhance the sensitivity of pancreatic cancer cells to gemcitabine, possibly via upregulation of ENT1 expression (Wang et al., 2011). It was thus of interest to compare I3C with the hits that we found. In our hands, however, I3C had no measurable effect on either the cell viability in the presence or absence of gemcitabine (nor on the expression profiles discussed below). This is entirely consistent with its low structural similarity to the other hits as indicated above.

### Effect of fragments on the growth of Panc1 cells

**Figure 10:**
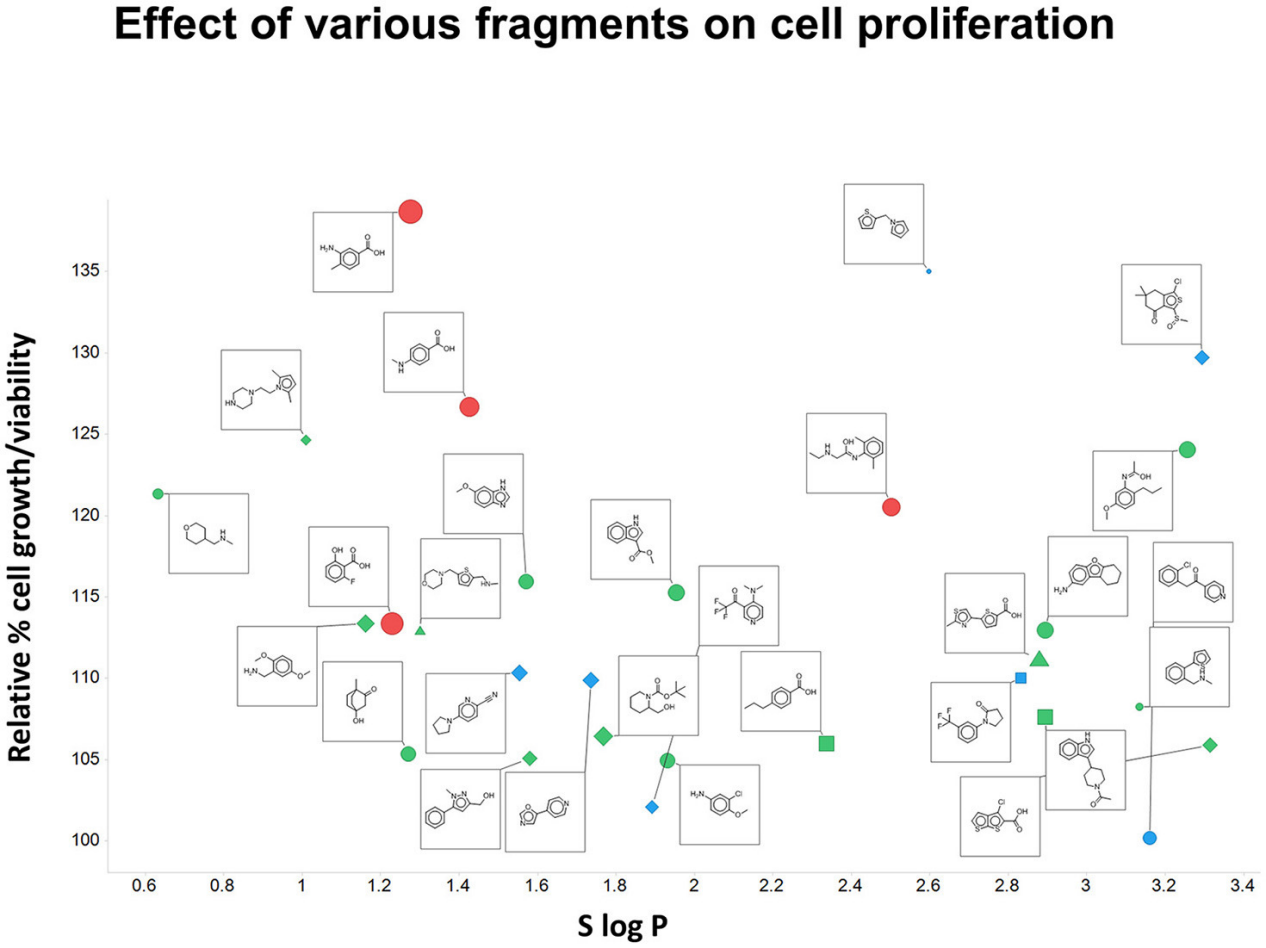
**Effect of various fragments on cell growth/viability relative to untreated controls**. Also plotted are the number of H-bond acceptors (by shape; square 1, circle 2, diamond 3, triangle 4), H-bond donors (by color, blue 0, green 1, red 2, yellow 3), total polar surface area (by size of symbol, up to 63 Å^2^) and S log P (on the abscissa).

Although this was not the main focus of the present paper, we did note (as mentioned above) that the fragments themselves could stimulate the growth of Panc1 cells relative to that of controls (as measured by OD). This is illustrated in Figure 10 for 28 of the fragments on which we focussed. Also encoded with the structures are the number of H-bond donors and acceptors, the total polar surface area of the fragments, and (on the abscissa) the S log *P*-values. It is clear (i) that virtually every fragment could stimulate the growth of the cells, and (ii) that there was no particularly obvious relationship of the extent of such stimulation with any of the descriptors stated.

### Effect of gemcitabine and fragments on the expression of selected transcripts in Panc 1 cells

**Figure 11:**
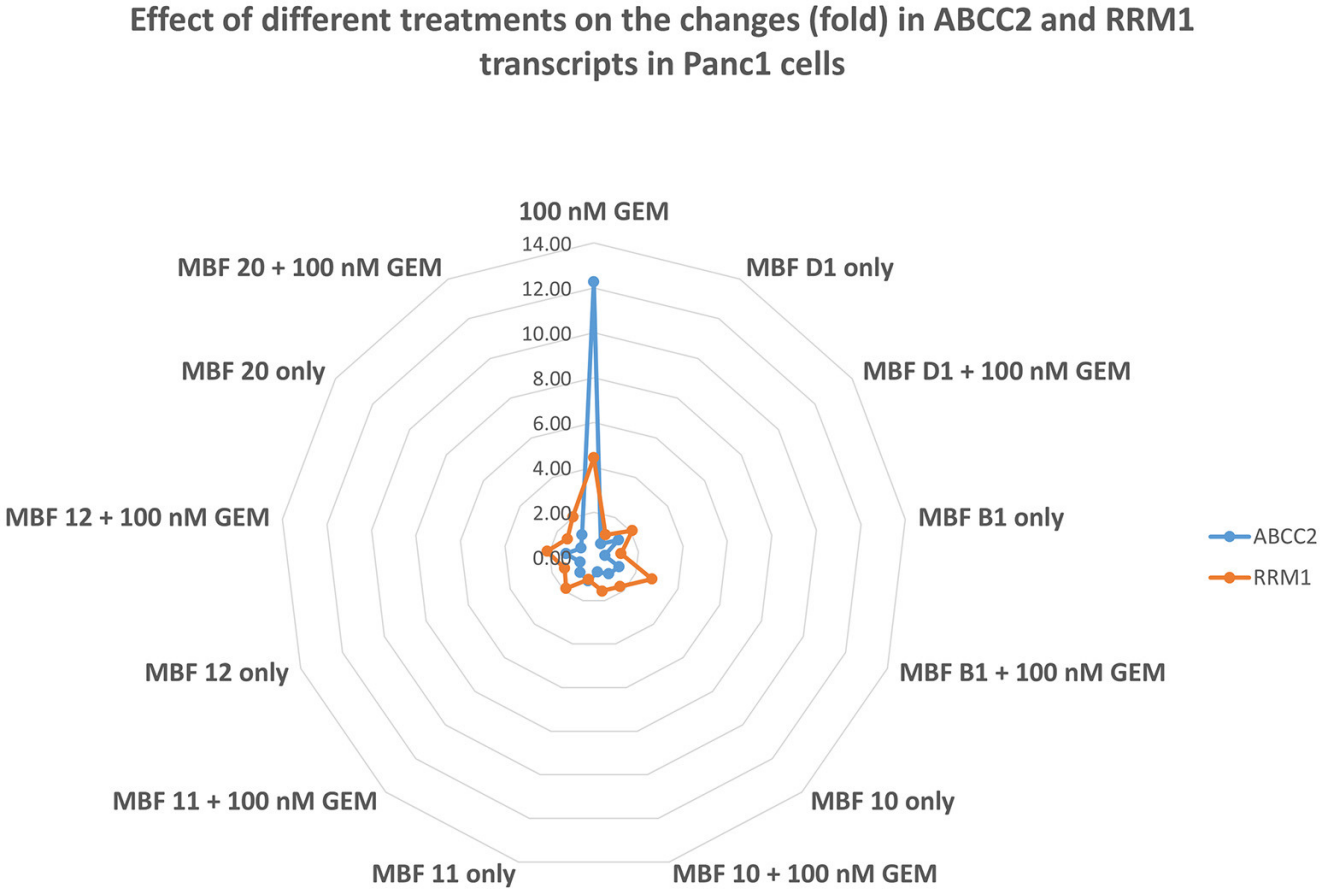
**Effect of gemcitabine ± various Maybridge fragments on the expression of transcripts for ABCC2 and RRM1**. Each experiment was performed three times, as described in Materials and Methods, and the mean is shown. For clarity, SD and statistical significance data are given only in the legends to Tables 3, 4.

Given that there was evidence that the fragments did not affect gemcitabine uptake directly, we assumed that they must be working by influencing the activity or expression of appropriate targets (and certainly small molecules can affect transporter expression, (e.g., Mrozikiewicz et al., 2014). To this end, we designed primers to enable PCR of transcripts relevant to gemcitabine transport and metabolism. Table 3 shows each of those that were detectable within 35 PCR cycles when treated (i) with gemcitabine alone, (ii) with Maybridge fragment D1 alone, and (iii) with both gemcitabine and D1. Strikingly, gemcitabine increases the expression of the ABCC2 efflux transporter (MRP2) more than 12-fold, and that of RRM1 more than fourfold, while the addition of D1 largely reverses both of these effects. It would seem that these are by far the largest contributors to the efficacy of fragment D1 in enhancing the cytotoxicity of gemcitabine, and the same is true for each of the other fragments (Table 4 and Figure 11). However, the ABCC2 inhibitor MK-571 (e.g., Weiss et al., 2007; Noma et al., 2008) at 20 μM had no effect on the viability of Panc1 cells treated with Gemcitabine alone (data not shown), possibly implying that RRM1 was the more significant contributor to the phenotypic changes in resistance.

**Table 3:** **Changes in the transcript level of relevant transporters and other genes when treated with gemcitabine and/or fragment D1**.

**Gene**	**Fold changes**		
	**Treatment with 100 nM GEM**	**Treatment with MBF D1 only**	**Treatment with MBF D1 and 100 nM GEM**
ENT1	0.87 ± 0.13	0.79 ± 0.12	1.08 ± 0.17
ENT2	0.57 ± 0.13	0.98 ± 0.27	0.59 ± 0.17
ENT3	2.58 ± 0.11	1.18 ± 0.64^*^	0.89 ± 0.20^***^
ABCC2	12.27 ± 0.34	0.66 ± 0.14^***^	1.33 ± 0.33^***^
ABCC3	0.16 ± 0.48	2.10 ± 0.09^**^	0.54 ± 0.18
ABCC4	0.53 ± 0.10	0.90 ± 0.23	0.36 ± 0.14
ABCC5	0.50 ± 0.11	1.18 ± 0.32^*^	1.21 ± 0.15^**^
ABCC10	1.61 ± 0.48	0.53 ± 0.08^*^	0.48 ± 0.16^*^
RRM1	4.43 ± 0.13	1.11 ± 0.17^***^	2.07 ± 0.16^***^

Only those transcripts detectable within 35 PCR cycles are shown. Data are given as mean ± standard deviation. A 2-sided T-test was performed to assess statistical significance against GEM alone, P-values being encoded as

* *< 0.05*,

** *< 0.01*,

*** *< 0.001*.

**Table 4:** **Changes in the transcript level of ABCC2 and RRM1 when treated with gemcitabine and/or the other fragment hits**.

	**Treatment**	**Gene fold changes**			
		**ABCC2**	**STDEV**	**RRM1**	**STDEV**
GEM	100 nM GEM	12.27	±0.34	4.43	±0.13
MBF D1	MBF D1 only	0.66^***^	±0.14	1.11^***^	±0.17
	MBF D1 + 100 nM GEM	1.33^***^	±0.33	2.07^***^	±0.16
MBF B1	MBF B1 only	0.49^***^	±0.08	1.22^***^	±0.12
	MBF B1 + 100 nM GEM	1.21^***^	±0.53	2.77^***^	±0.11
MBF 10	MBF 10 only	1.00^***^	±0.23	1.76^***^	±0.14
	MBF 10 + 100 nM GEM	0.68^***^	±0.05	1.56^***^	±0.08
MBF 11	MBF 11 only	1.09^***^	±0.09	1.04^***^	±0.11
	MBF 11 + 100 nM GEM	0.93^***^	±0.15	1.88^***^	±0.20
MBF 12	MBF 12 only	0.65^***^	±0.13	1.39^***^	±0.09
	MBF 12 + 100 nM GEM	1.25^***^	±0.19	2.11^***^	±0.15
MBF 20	MBF 20 only	0.7^***^	±0.06	1.43^***^	±0.11
	MBF 20 + 100 nM GEM	1.13^***^	±0.05	2.02^***^	±0.12

A 2-sided T-test was performed to assess statistical significance vs. GEM alone, P-values being encoded as * < 0.05, ** < 0.01,

*** *< 0.001*.

### Selectivity of fragments for increasing transporter expression

**Figure 12:**
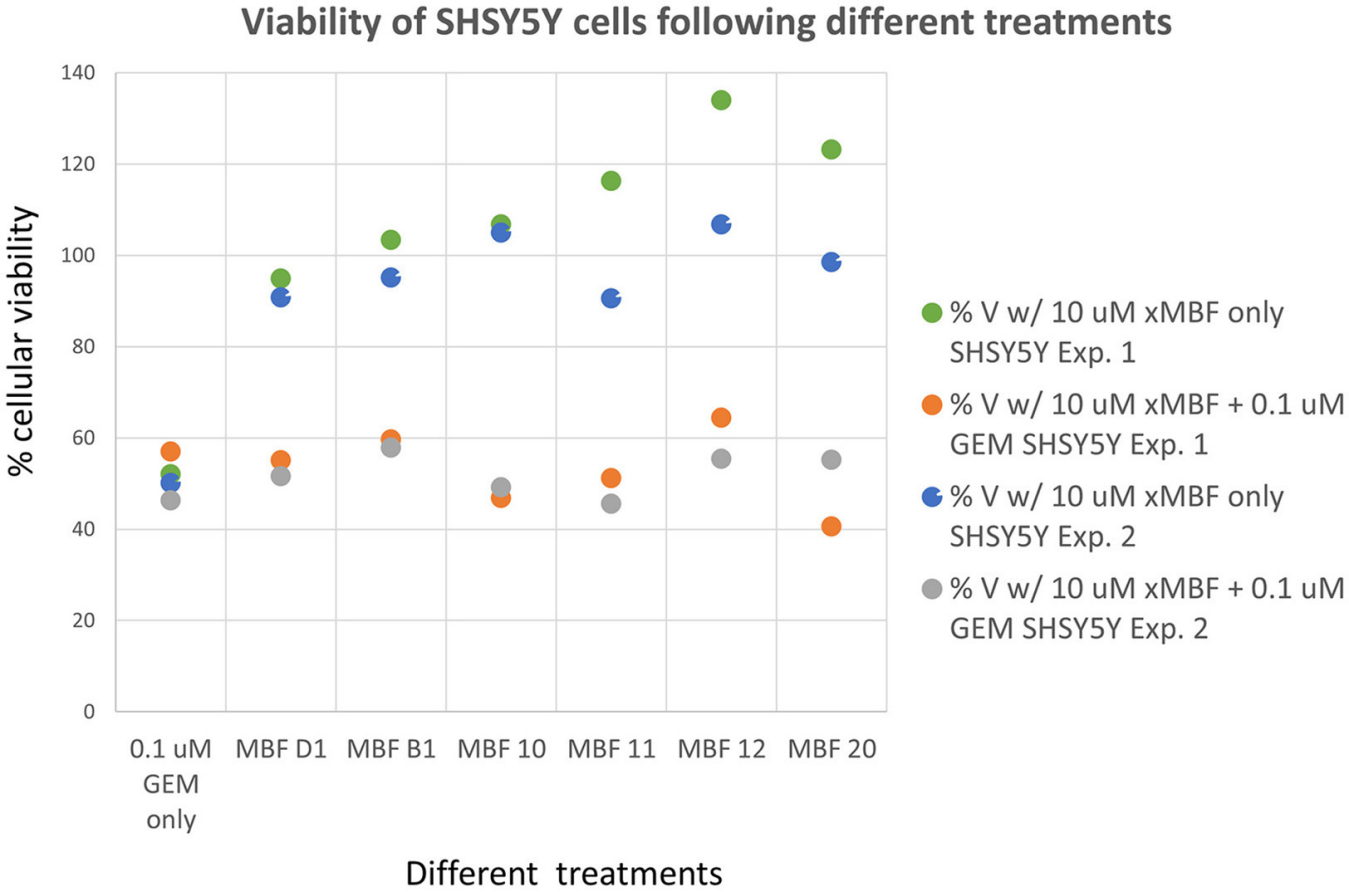
**Effects of gemcitabine and gemcitabine plus fragments on the viability of SH-SY5Y cells**. Apart from fragments 10 and 20, the effects of the fragments were not statistically significant at the *P* < 0.05 level, *n* = 3 per experiment.

**Figure 13:**
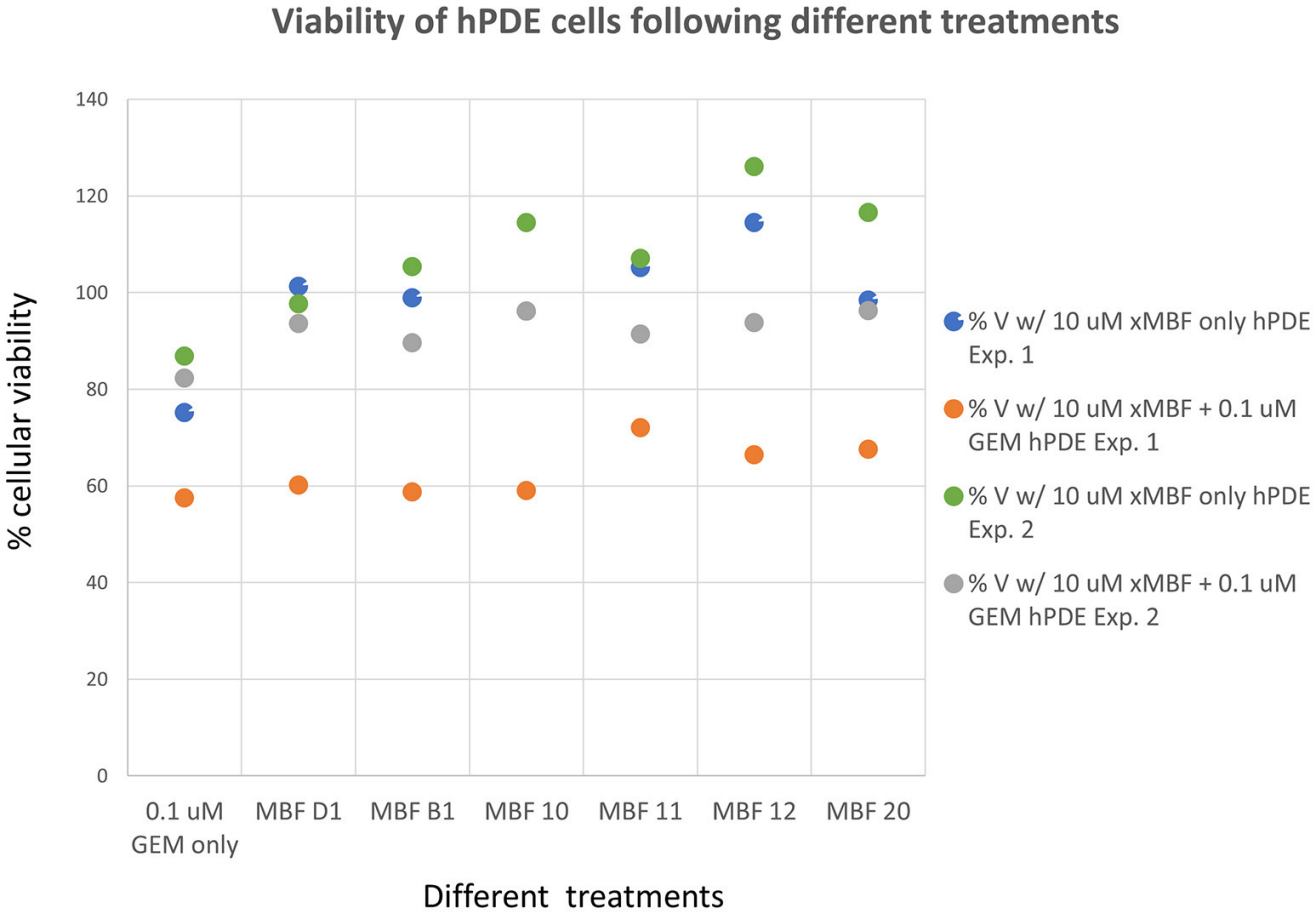
**Effects of gemcitabine and gemcitabine plus fragments on the viability of hPDE cells**. The effects of the fragments were not statistically significant at the *P* < 0.05 level, *n* = 3 per experiment.

**Figure 14:**
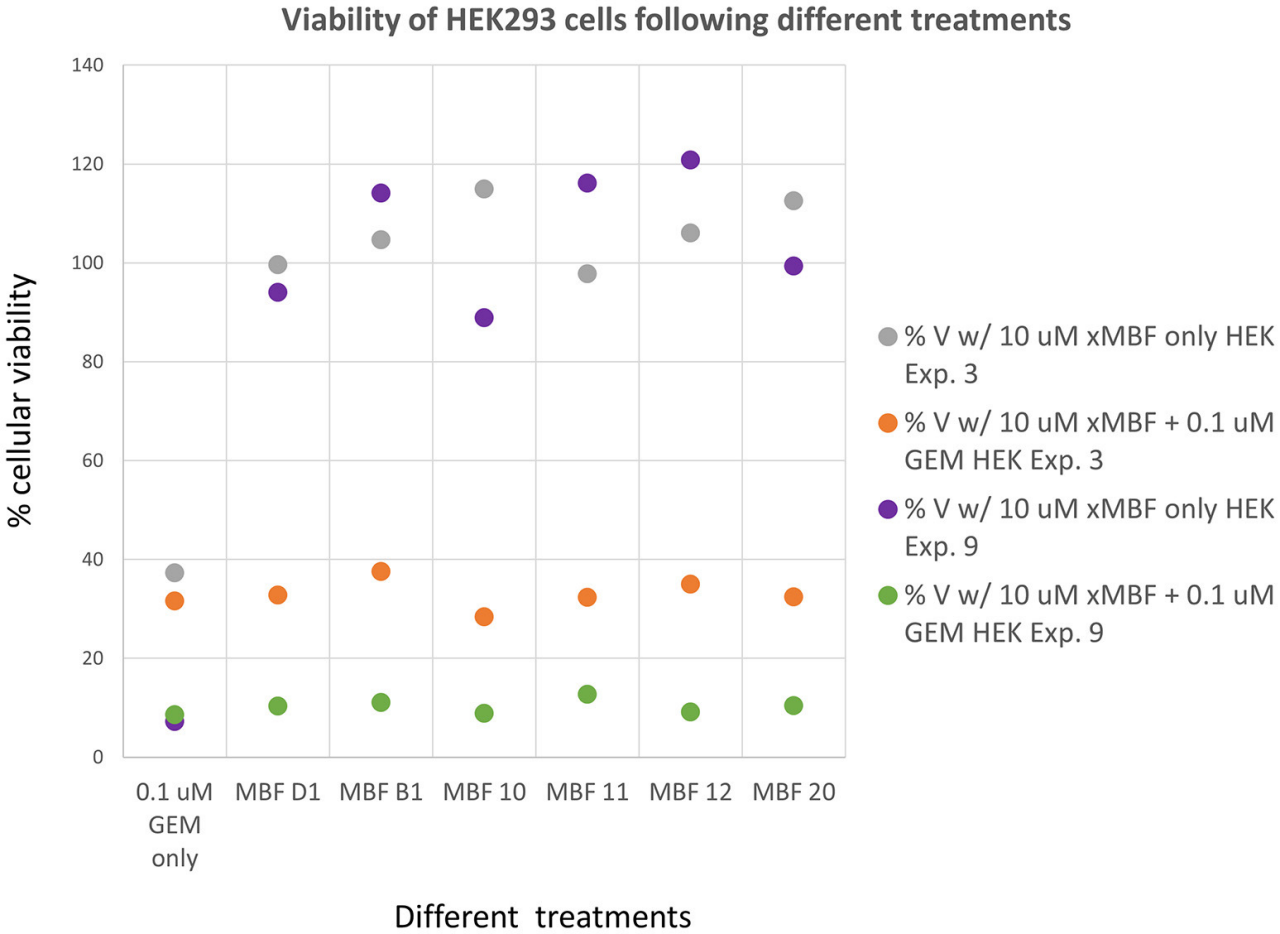
**Effects of gemcitabine and gemcitabine plus fragments on the viability of HEK293 cells**. Experiments were performed as described, and as per the legend to Figures 3, 8. The effects of the fragments were not statistically significant at the *P* < 0.05 level, *n* = 3 per experiment.

Having seen that various of the fragments could increase the toxicity of gemcitabine to Panc1 cells, it was of interest to see whether this was a cell-selective phenomenon. Although time did not permit an exhaustive study, we noted that fragments 10 and 20 also had these toxicity-enhancing effect for the neuroblastoma SH-SY5Y cell line while B1, D1, 11, and 12 did not (Figure 12). No fragments seemed to have any such effects on the non-cancerous pancreatic cell line hPDE (Figure 13) and HEK293 cells (Figure 14), implying that there is or can be at least some degree of specificity in our “binary weapon” approach. Clearly a larger-scale study (including both larger libraries and more cell lines) would be able to discover molecules with both potency and selectivity.

## Discussion

**Figure 15:**
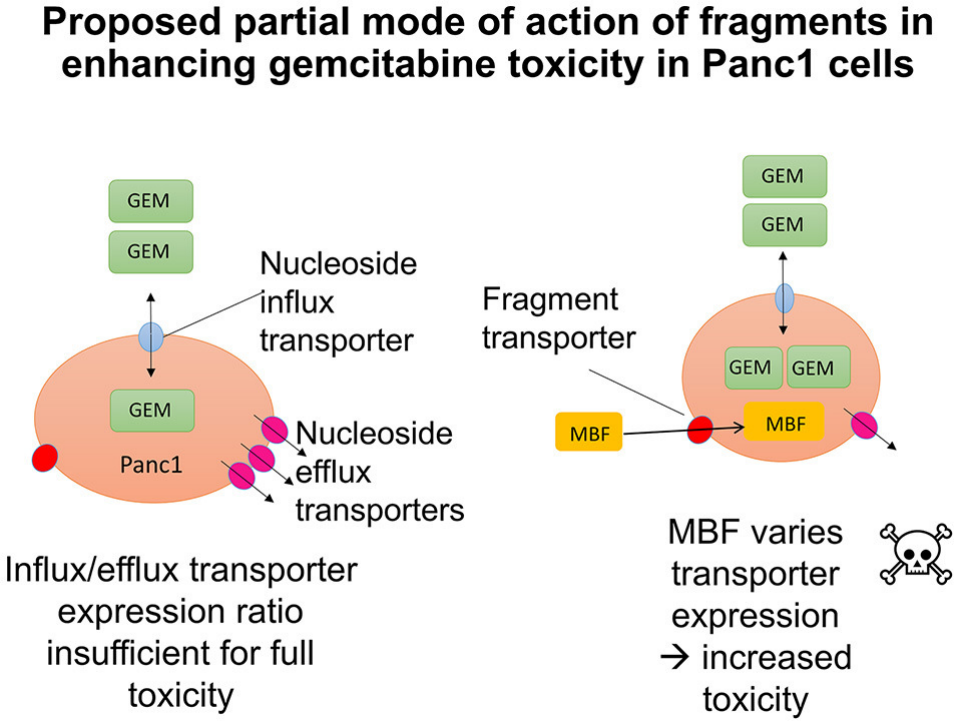
**Cartoons illustrating the potential modes of action of fragments in enhancing transporter-mediated gemcitabine toxicity in Panc1 cells**. (The smaller effects on RRM1 are ignored for clarity). **Left:** Original hypothesis that fragments would stimulate the activity of uptake transporters. **Right:** Actual mechanism based on PCR data.

In the present work, we sought to develop the idea that we might affect the transporter-mediated disposition of small-molecule drugs via the addition of a second small molecule that of itself had no inhibitory pharmacological effect but that influenced the expression of transporters for the primary drug (Figure 15). We refer to this as a “binary weapon” strategy. The specific phenotypic effect we sought was for a molecule that on its own had no such effect to increase the toxicity of the nucleoside analog gemcitabine to Panc1 pancreatic cancer cells (Figures 1–3).

Given the recognition (O'Hagan and Kell, 2015c) that more some 25% of marketed drugs are in fact no larger than the polar “rule-of-three”-compliant (Congreve et al., 2003) molecules used in fragment-based drug discovery, we used an initial screen of a 500-member polar drug fragment library. This yielded three “hits” (Figures 4, 5). The structures of 20 of the other 2000 members of this library had a Tanimoto similarity greater than 0.7 to those of the initial hits, and each was itself a hit (Figure 6) (with the cheminformatics thus providing for a massive enrichment in the fraction of successful experiments). We chose the top six representatives for further study. They each bore reasonable structural similarities to each other (two were in fact isomers), lending strength to the self-consistency of both our conceptual and experimental strategies (Figures 7, 8).

Existing literature had suggested that indole-3-carbinol might play a similar role to that of our fragments, but in our hands it was without effect, and nor was it structurally similar to any of our hits (Figure 9). We therefore discounted it.

There is an interesting issue when the phenotypic activity being measured is in fact cell death, as it is then impossible legitimately to compare bulk measurements of biochemical changes with individual-cell viabilities. This is because with bulk or ensemble measurements one does not know if say a lowering of a biochemical parameter by 50% means that all of the cells have lost half of it or half of the cells have lost all of it (or anything in between) (Kell et al., 1991, 1998; Davey and Kell, 1996). In the event, the mechanism was very clear, however.

Because the fragments were themselves without negative effects on the cells in the absence of gemcitabine (interestingly, many of them actually stimulated cell growth, Figure 1, so each had to be compared to the appropriate control!), we next designed suitable primers to assess the expression levels of all the candidate transporters plus ribonucleotide reductase. In our hands, only the ENT1-3 uptake and ABCC2,3,4,5, and 10 efflux transporters displayed measurable transcripts, along with RRM1. Very strikingly, the addition of gemcitabine alone increased the expression of the transcript for ABCC2 (MRP2) by more than 12-fold, and that of RRM1 by more than fourfold, and each of the fragment “hits” served to reverse this, at least in part (Figure 11). The effects on ABCC2 are thus consistent with the finding (Horiguchi et al., 2013) that it may be a major efflux pump for gemcitabine.

It seems, therefore, that while the effect was here mediated more by efflux than influx transporters, the binary weapon idea is hereby fully confirmed: our results show that it is possible to find molecules that manipulate the expression of transporters that are involved in the bioactivity of a pharmaceutical drug, and that there is a certain degree of specificity in this for pancreatic cancer cells (Figures 12–14). This could explain, at least in part, the basis for the selective toxicity of a drug that is otherwise cytotoxic generally (Figure 15). The next steps will involve determining much more extensively how much any such activity differs, or can be made to differ (as do most transcript levels), between different cells.

## Author contributions

DK and PD designed the study. All the experimental work was performed by JG, who was supervised by PD and DK. Some of the cheminformatics analyses were performed by SO. All authors contributed to and approved the writing of the manuscript.

### Conflict of interest statement

The authors declare that the research was conducted in the absence of any commercial or financial relationships that could be construed as a potential conflict of interest.
